# Dietary Nonenriched and Iron-Enriched Yeasts Improve Hematological and Antioxidant Parameters in Rainbow Trout, *Oncorhynchus mykiss*, Fed on Diets Containing Cottonseed Meal

**DOI:** 10.1155/anu/9955172

**Published:** 2025-04-06

**Authors:** Seyyed Morteza Hoseini, Esmail Pagheh, Abbassali Aghaei Moghaddam, Behrouz Gharavi, Melika Ghelichpour

**Affiliations:** Inland Waters Aquatics Resources Research Center, Iranian Fisheries Sciences Research Institute, Agricultural Research, Education and Extension Organization, Gorgan, Iran

**Keywords:** antioxidant, cottonseed meal, gossypol, iron, yeast

## Abstract

This study aimed to evaluate the effects of dietary cottonseed meal (CSM) as a partial substitute for soybean meal, along with iron-enriched and nonenriched *Saccharomyces cerevisiae* (PTCC 5052) on growth performance, anemia, iron and gossypol retention, and hepatic antioxidant and histological characteristics in juvenile rainbow trout, *Oncorhynchus mykiss*. Fish (31.6 ± 0.33 g) were distributed across 16 tanks in four quadruplicate treatment groups. A control diet without CSM, a diet containing 15% CSM (CSM), a diet with 15% CSM plus 1 × 10^8^ cfu/g of *S. cerevisiae* (CSMY), and a diet with 15% CSM plus 1 × 10^8^ cfu/g of iron-enriched *S. cerevisiae* (CSMYFE) were fed to the treatment groups. After 8 weeks of feeding, there were no significant differences in growth performance, feed efficiency, total/differential leukocyte counts, plasma iron concentration, alanine aminotransferase, aspartate aminotransferase, and hepatic antioxidant and histological characteristics among the treatment groups. Blood erythrocyte counts and hematological indices were similar across treatments, but the CSM group exhibited significantly lower blood hematocrit (*p* = 0.005) and hemoglobin (*p* = 0.002) levels compared to the other treatments. Hepatic iron concentration was significantly higher in the CSMYFE group than in the other treatments (*p* = 0.001). Hepatic gossypol concentrations in the CSM, CSMY, and CSMYFE treatments were similar and significantly higher than that of the control treatment (*p*  < 0.001). Plasma total antioxidant capacity (*p* = 0.002) and ascorbate level (*p* = 0.025) were significantly elevated in the CSMY and CSMYFE groups compared to the other treatments. In conclusion, a dietary inclusion of 15% CSM as an alternative to soybean meal does not negatively affect fish growth performance, hepatic histology, or antioxidant parameters; however, it does result in decreased hematocrit and hemoglobin levels while increasing hepatic gossypol levels. The inclusion of *S. cerevisiae*, whether iron-enriched or nonenriched, mitigates the decreases in blood hematocrit and hemoglobin levels and enhances hepatic antioxidant parameters.

## 1. Introduction

Cottonseed, a nutrient-rich byproduct obtained after the extraction of cotton fiber, has garnered attention as a potential alternative to soybean meal in fish diets [1]. In the 2023/2024 production year, global cottonseed production surpassed 41 million metric tons [2]. This byproduct contains high amounts of protein (33%–41%) and fat (17%–22%) [3, 4], making it appealing for aquaculture. However, there are limitations in using this product in aquaculture. It contains gossypol, an antinutrient that binds iron and rendering it unavailable for absorption [5]. Gossypol is particularly toxic to monogastric animals, and excessive inclusion of cottonseed meal (CSM) in fish diets can elevate free gossypol levels, negatively impacting fish growth [6, 7]. Moreover, CSM has lower levels of the essential amino acid lysine compared to soybean meal [8], which necessitates the amino acid supplementation in diets containing CSM. Additionally, CSM higher fiber content compared to soybean meal [9, 10], which may pose challenges for carnivorous species such as rainbow trout, *Oncorhynchus mykiss*, which require protein-rich diets.

Despite the problems stated above, CSM has been used in diets of various species [11]. Research indicates that rainbow trout can grow from ~11 g to 40 g on a diet containing 10% CSM (as an alternative to fishmeal and equating to 110 mg/kg of free gossypol) without adversely affecting fish growth or feed efficiency [12]. However, adding 15% of the same CSM to the diet (containing 165 mg/kg free gossypol) significantly decreased feed efficiency and protein retention. Furthermore, when reared from about 40 g to 100 g, incorporating 15.2% low-gossypol CSM (with a free gossypol level of 10 mg/kg) also showed no negative impacts on the fish growth and feed efficiency [13]. Studies on adult rainbow trout suggest that CSM proportions can be increased up to 58.8% without detrimental effects on growth performance and feed efficiency [14–16]. Nonetheless, this high inclusion level has been associated with decreased reproductive performance and progeny quality, as well as signs of anemia in the fish. Given the limited data on the effects of dietary CSM on various health aspects of juvenile rainbow trout and strategies to enhance the efficacy of CSM-containing diets in this species, further research is warranted.

Probiotics have emerged as a beneficial addition in aquaculture, enhancing growth, immunity, and antioxidant capacity in fish [17]. Their application in plant-based diets has been demonstrated to improve growth performance and overall health across various fish species [18–20]. *Saccharomyces cerevisiae* is one of the most commonly utilized yeasts in aquaculture settings [21]. Numerous studies have demonstrated that *S. cerevisiae*, whether in whole cell form, as a yeast extract, or as cell wall components, can enhance growth, immunity, and disease resistance in various fish species [22–29]. Notably, this yeast has been shown to neutralize gossypol from CSM under in vitro fermentation conditions by up to 870% [30]. Fermentation process has been recognized in the gut of rainbow trout [31, 32]; hence, incorporating *S. cerevisiae* as a fermentation agent in fish feed may effectively lower gossypol levels while simultaneously promoting fish growth and health.

Alternatively, dietary iron supplementation offers another method for neutralizing gossypol from CSM. Iron binds with free gossypol, rendering it unavailable for absorption [33]. Research across various fish species indicates that adding iron salts to gossypol-containing diets can reduce gossypol accumulation in body tissues and mitigate its adverse effects [5, 34]. However, excessive dietary iron can lead to negative outcomes, such as increased susceptibility to diseases [35]. Organic forms of iron have high bioavailability, which decrease the required iron in the fish diet [36]. These organic salts are produced by adding iron salts to the culture medium of specific microbes, which convert them into organic forms via amino acids like methionine [37]. So, it is worth assessing if organic iron benefits fish fed on diets containing gossypol.

The use of organic iron in the diets of mirror carp, *Cyprinus carpio* var. *specularis* [38]; channel catfish, *Ictalurus punctatus* [39, 40]; cobia, *Rachycentron canadum* [41]; and gilthead bream, *Sparus aurata* [42, 43] has been shown to lower dietary iron requirements and enhance growth and immune indices. However, the impact of iron-enriched yeast on gossypol neutralization and its effects on the growth and health of rainbow trout remain unexplored. Therefore, this study aims to investigate the effects of replacing soybean meal with CSM and incorporating iron-enriched *S. cerevisiae* on gossypol neutralization, growth performance, and hemato-biochemical parameters, as well as histological and antioxidant parameters of the liver in juvenile rainbow trout.

## 2. Materials and Methods

### 2.1. Ethics

This study was approved by the Scientific Committee of the Inland Waters Aquatics Resources Research Center, Iran (No.: 1402/IWASRC-9; date December 6, 2023).

### 2.2. *S*. *cerevisiae* Culture and Enrichment

Lyophilized *S. cerevisiae* (PTCC 5052) was obtained from the Iranian Research Organization for Science and Technology in Tehran, Iran. The yeast was initially cultured on potato dextrose agar medium (Q-Lab Co., Ohio, USA) for 24 h at 25°C. Following the protocol outlined by Gaensly et al. [44], iron enrichment was conducted in potato dextrose broth (PDB) medium (Q-Lab Co., Ohio, USA). In brief, 500 mL of PDB was prepared in a 1-L Erlenmeyer flask and inoculated with *S. cerevisiae*. The flask was incubated with shaking at 100 rpm and 25°C for 48 h, until the optical density of the suspension reached ~1.600 at 600 nm. At this point, the nonenriched yeast was harvested, while iron inoculation proceeded to create the enriched yeast. For this, 497 mg/L of ferrous sulfate was added to the culture medium, and the flask was further incubated under the same conditions for an additional 12 h. After this period, the iron-enriched yeast was harvested. Both types of yeast were stored in physiological saline at 4°C for up to 24 h prior to their incorporation into fish diets. The iron content in the nonenriched and enriched yeasts was measured at 0.07 mg/g and 18.9 mg/g dry weight, respectively.

### 2.3. Diets, Fish, and Experimental Protocol

Feed ingredients were sourced from local markets, and diets were formulated using the Windows User-Friendly Feed Formulation Excel spreadsheet. A control diet was created without CSM and contained 250 g/kg soybean meal. Three experimental diets were prepared by decreasing soybean meal to 120 g/kg and adding 150 g/kg of CSM: one without yeast supplementation (CSM), one supplemented with 1 × 10^8^ cfu/g of nonenriched yeast (CSMY), and another with 1 × 10^8^ cfu/g of iron-enriched yeast (CSMYFE). Viable dietary yeast cells were determined every other week by culturing on PDA medium and found to be 0.82 × 10^7^ to 1 × 10^8^ cfu/g for CSMY and 0.78 × 10^7^ to 1.1 × 10^8^ cfu/g for CSMYFE. The inclusion level of dietary yeast was based on a previous study on rainbow trout [29]. The level of dietary CSM was chosen based on a previous study on rainbow trout, showing dietary CSM above 100 g/kg resulted in decrements in fish growth and dietary protein efficiency ratio (Cheng and Hardy, 2002). Moreover, higher levels of dietary CSM can be achieved by incorporating concentrated protein sources (e.g., fishmeal, casein, and gluten), which increase feed price and decrease sustainability. Chemical composition and amino acid and fatty acid profiles of the diets are shown in Tables 1 and 2. To prepare the diets, the feed ingredients were thoroughly mixed for 15 min. Subsequently, 0.35 L of water was added to the mixture, along with the yeast culture, which had been premixed with the water. The combined ingredients were then mixed for an additional 10 min to form a paste, which was subsequently pelleted using a meat grinder.

A total of 240 healthy fish, all of similar size, were acquired from a private farm in Sari, Iran. These fish were distributed across 16 plastic tanks, each with a capacity of 300 L but containing 120 L of water, at the Fisheries Research Station of Gharasoo in Torkman, Iran. Each tank housed 15 fish, averaging ~31.6 ± 0.33 g. The tanks were connected to a well water supply system, maintaining a flow rate of 1 L/min throughout the experiment. Aeration was provided by a central pump, and daily waste siphoning was performed from the tank bottoms. Additionally, the internal surfaces of the tanks were cleaned twice a week. The fish were fed specific diets for a duration of 8 weeks, with a daily feeding rate of 3%–4% of total biomass, divided into two meals. Each diet was allocated to four tanks, and feed amounts were adjusted biweekly based on the biomass in each tank. During the experiment, water quality parameters were consistently monitored: temperature (13.3 ± 2.5°C), dissolved oxygen (8.57 ± 1.00 mg/L), pH (8.00 ± 0.04), unionized ammonia (0.01 ± 0.001 mg/L), and iron levels (94.5 ± 4.21 µg/L). Measurements were taken using a digital probe (HQ40D, Loveland, Colorado, USA) and a customized photometer (Model 7100, Palintest House, Gateshead, UK).

At the conclusion of the rearing period, three fish from each tank were captured and anesthetized in a clove solution (2 g/L). Blood was collected using a heparinized syringe inserted behind the anal fin and was divided into two portions: one for hematological analysis and the other for plasma separation. Plasma was obtained by centrifuging the blood for 10 min at 4°C and was subsequently stored at −70°C for future analysis. Following blood collection, the fish were euthanized via spinal cord severance. The abdominal cavity was then opened to collect a liver sample, which was immediately frozen in liquid nitrogen for antioxidant parameter measurement. Additionally, another segment of the liver was dissected and fixed in 10% formalin for histological examinations.

### 2.4. Analysis of Diets

The chemical composition analysis of the diets was conducted according to AOAC [45]. The samples were dried at 105°C for 24 h to determine moisture content. The protein content of the samples was measured using the Kjeldahl method, calculating nitrogen content. The obtained nitrogen value was multiplied by a factor of 6.25 to determine protein content. The fat content of the diet was determined using the ether extraction method and a Soxhlet apparatus, using petroleum ether as the solvent. For ash determination, samples were burned at 550°C for 8 h.

To determine the amino acid profile, an HPLC system equipped with a fluorescent detector (Agilent 1090 system, Palo Alto, CA, USA) was used. Initially, the sample was digested with 6N hydrochloric acid at 115°C for 24 h. The resulting solution was filtered through a syringe filter, followed by protein precipitation (using trichloroacetic acid) and derivatization (using OPA). The amount of each amino acid was determined based on its standard peak and retention time on the C18 column.

Total lipids from the experimental diets were extracted through sample homogenization in a chloroform/methanol solution (2:1, v/v), following the protocols outlined by Folch, Lees, and Stanley [46]. Methyl esters were generated via transmethylation using methanolic potassium hydroxide and n-heptane as described by Agh, Jasour, and Noori [47]. The fatty acid composition was determined using an autosampler gas chromatography (GC) (Agilent Technologies 7890N, USA), which was equipped with a flame ionization detector and a cyanopropyl-phenyl capillary column (DB-225MS, 30 m × 0.250 mm ID × 0.25 μm film thickness, USA). Identification of the fatty acids was achieved by comparing their retention times to those of an external commercial standard mixture (GLC-68d, Nu-Chek Prep., MN, USA).

### 2.5. Growth Performance Evaluation

Fish final weight and feed intake throughout the experiment were used for the following calculations:

  $$\begin{array}{c} \text{Specific growth rate }\left(\%/\text{day}\right)=100\times \frac{\ln\;\left(\text{final weight}\right)\text{ − }\ln\;\left(\text{initial weight}\right)}{\text{Rearing period }\left(\text{days}\right)}, \end{array}$$

  $$\begin{array}{c} \text{Weight gain }\left(\%\right)=100\times \frac{\text{Final weight}-\text{initial weight}}{\text{Initial weight}}, \end{array}$$

  $$\begin{array}{c} \text{Feed conversion ratio}=\frac{\text{Feed intake}}{\text{Final weight}-\text{initial weight}}. \end{array}$$

### 2.6. Hematological Assessments

Hematological parameters were determined based on the method of Dacie and Lewis [48]. Dacie solution was used to dilute blood samples before the blood cell being counted under a microscope using a Neubauer chamber. Mean corpuscular volume (MCV), mean corpuscular hemoglobin (MCH), and mean corpuscular hemoglobin concentration (MCHC) were calculated according to Dacie and Lewis [48]. For the differential leukocyte count, blood samples were fixed on slides and stained with Giemsa. Lymphocyte, neutrophil, and monocyte were counted based on their morphological characteristics under the microscope. Hematocrit was measured using a microcentrifuge, and hemoglobin levels were determined using a biochemical diagnostic kit (Zist Chem Co., Tehran, Iran).

### 2.7. Liver Antioxidant Parameters

Liver samples were homogenized in a 1:1 ratio with phosphate buffer (pH 7.0) and then centrifuged for 15 min at 4°C (13,000 × *g*). The supernatant was collected and stored at −70°C until antioxidant indices were measured. The activities of superoxide dismutase (SOD) and catalase (CAT), as well as the levels of ascorbate, total antioxidant capacity (TAC), reduced glutathione (GSH), and malondialdehyde (MDA), were measured using commercial kits from ZellBio GmbH (Ulm, Germany). To calculate the specific activity of these enzymes, the soluble protein concentration of the samples was measured using a microprotein kit (Zist Chem Co., Tehran, Iran).

### 2.8. Measurement of Plasma, Liver, and Dietary Iron Concentrations

Plasma iron levels were measured using a medical diagnostic kit from Darman-Faraz-Kav Co. (Tehran, Iran) based on the manufacturer's instructions. The detection limit of the kit was 5 µg/dL, and the detection range was up to 800 µg/dL (linear *R*^2^ = 0.995). The validity of the kit for trout plasma samples was checked by dilution and spiking with a recovery range of 98.5%–103%.

Diet and liver samples were dried in an oven at 70°C, followed by digestion at 40°C in nitric acid (65%) at a ratio of 15:1 (v:w). Then, the iron concentrations in the digested samples were determined by ferrozine method [49]. In brief, 300 μL of the digested samples was mixed with 30 μL of a reagent containing 0.0065 M ferrozine, 2.5 M ammonium acetate, 0.0065 M neocuproine, and 1 M ascorbic acid. The mixture was incubated at room temperature for 30 min, and its absorbance was recorded at 550 nm. The iron contents in the samples were calculated using FeCl_3_ as standard.

### 2.9. Measurement of Free and Total Gossypol in the Liver and Diets

To quantify free and total gossypol in the diets and liver samples, an extraction process was employed. Free gossypol was extracted using a mixture of 2-propanol and hexane, while total gossypol was extracted with dimethylformamide. Subsequently, aniline was added to convert the gossypol in the samples into dianiline gossypol, which forms a yellow complex that exhibits maximum absorbance at a wavelength of 440 nm. The intensity of the resulting color is directly proportional to the concentration of gossypol present in the sample [50].

### 2.10. Preparation of Tissue Sections From Fish Liver

For tissue section preparation, two fish from each treatment group were anesthetized using a clove solution. Following anesthesia, the fish were euthanized via spinal cord severance, and a piece of the liver (apical part of the organ with ~0.5 cm diameter) was dissected and fixed in 10% buffered formalin. The samples underwent dehydration and paraffin embedding using a tissue processor, resulting in paraffin blocks. Using a microtome, sections of 5 µm in thickness were prepared, mounted on slides, and stained with hematoxylin and eosin. Two sections were prepared from each sample, spaced 200 µm apart. These prepared tissue sections were then examined microscopically to assess histological changes in the liver.

### 2.11. Statistical Analysis

The normality of the data was first confirmed using the Shapiro-Wilk test. The significant effects of dietary treatments on various parameters—including fish growth, hepatic antioxidant levels, plasma and liver iron concentrations, hepatic gossypol levels, hematocrit, hemoglobin, MCHC, and leukocyte counts—were analyzed using one-way ANOVA followed by Duncan's test. For erythrocyte count, MCV, and MCH, nonparametric tests (Kruskal–Wallis and Mann–Whitney *U* tests) were employed due to the lack of homogeneity of variance as indicated by Levene's test. Additionally, the percentage abundance of lymphocytes, monocytes, and neutrophils was arcsin-transformed before analysis with one-way ANOVA and Duncan's test. All statistical analyses were conducted using SPSS v22 software, with significance determined at a threshold of *p*  < 0.05.

## 3. Results

Growth performance and feed efficiency of the fish in different treatments are shown in Table 3. Accordingly, dietary treatments had no significant effects on the fish final weight, specific growth rate, weight gain, survival, and feed conversion ratio.

Hematological parameters are shown in Table 4. Dietary treatments significantly affected the blood hematocrit and hemoglobin levels. These parameters significantly decreased in the CSM treatment, compared to the control; however, dietary yeast and iron-enriched yeast supplementation restored these changes. Nevertheless, dietary treatments had no significant effects on the blood erythrocyte, MCV, MCH, MCHC, leukocyte, lymphocyte, neutrophil, and monocyte.

Dietary treatments had no significant effects on plasma iron concentrations but significantly affected hepatic iron and gossypol concentrations (Table 5). Hepatic iron concentration in CSMYFE treatment was significantly higher than the other treatments. There was no detectable gossypol in the liver of the control fish, but 38.3–41 µg/g gossypol was observed in the liver of the other treatments.

There were no significant differences in the hepatic SOD, CAT, GSH, and MDA levels among the treatments (Table 6). However, dietary treatments significantly affected the plasma TAC and ascorbate concentrations (Table 7). CSMY and CSMYFE had similar plasma TAC and ascorbate levels, significantly higher than those of the control and CSM treatments. Dietary treatments did not significantly affect the plasma ALT and AST activities (Table 7).

**Figure 1:**
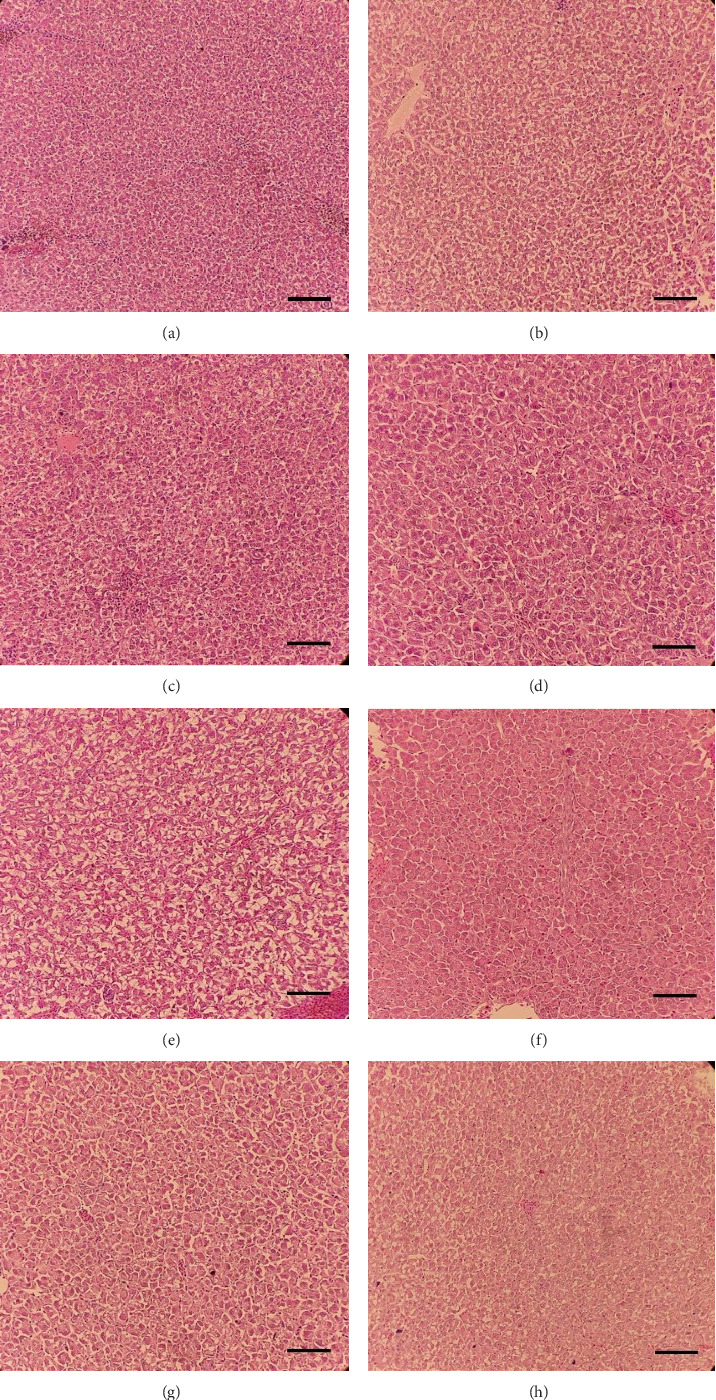
Representatives of the liver histological sections in the control (A and B), CSM (C and D), CSMY (E and F), and CSMYFE (G and H) treatments. The scale bars equal 25 µ (magnification 200×). Consider fat the mild fat deposition in some slides (eight out of eight samples in the control, two out of eight samples in the CSM, three out of eight samples in the CSMY, and five out of eight samples in the CSMYFE).

Histological sections of the fish liver are presented in Figure 1. Generally, the liver had normal structure and appearance in all treatments, except mild fat depositions in some samples. This lesion was observed in eight out of eight samples in the control, two out of eight samples in the CSM, three out of eight samples in the CSMY, and five out of eight samples in the CSMYFE.

## 4. Discussion

This study demonstrated that including 15% CSM in the diet of juvenile rainbow trout did not negatively impact growth rates or feed efficiency when the fish were raised from ~30 g to around 125 g. These results align with previous research that utilized low-gossypol CSM in diets for rainbow trout growing from about 40 g to 100 g [13]. Collectively, these findings suggest that dietary gossypol may not be a critical factor limiting the inclusion of CSM in the diets of rainbow trout within this weight range, especially since the current diets contained ~12 times higher gossypol levels than that in the earlier study. Supporting this theory, feeding on CSM-containing diets (15%–29%; 260–520 mg/kg free gossypol) led to numerically higher growth performance in tilapia, *Oreochromis* sp., despite lower phosphorus, copper, and magnesium digestibility and gossypol load in the fish liver [51]. On the other hand, when a diet containing 15% CSM (165 mg/kg of free gossypol) was administered to rainbow trout with an initial weight of 11 g, significant reductions in growth performance, feed efficiency, and protein retention were observed [12]. The observed differences in outcomes can be attributed to variations in dietary formulations. Cheng and Hardy [12] incorporated CSM into trout diets by replacing fishmeal, a strategy that may yield more significant effects (nutrient digestibility and antinutrient factors) compared to substituting it for soybean meal. Additionally, fish weight, which is likely related to their early life stage, could impact the permissible levels of dietary CSM. This hypothesis is reinforced by research on adult rainbow trout, which demonstrated that CSM levels as high as 58.8% did not hinder growth [14–16].

Supplementation with dietary yeast or iron-enriched yeast did not significantly affect growth performance or feed efficiency, in the present study. Supporting these results, dietary yeast supplementation had no benefits on growth performance of Nile tilapia, *Oreochromis niloticus* [52], and gibel carp, *Carassius auratus gibelio* [53]. In contrast, Zargham et al. [54] reported that *S. cerevisiae* (PTCC 5052) at a concentration of 1 × 10^8^ cfu/g improved survival and final weight in rainbow trout larvae. The discrepancies in these findings may be attributed to variations in fish weight and dietary composition, warranting further investigation. It is also important to consider that the potential benefits of dietary yeasts might be obscured by the absence of detrimental effects from dietary CSM on fish growth. Thus, the advantages of yeast supplementation may become more apparent when higher levels of CSM adversely affect fish growth, particularly in smaller fish.

Dietary inclusion of CSM has been linked to anemia in adult rainbow trout, characterized by reductions in blood hematocrit and hemoglobin levels [14, 55]. The present study is the first to demonstrate that CSM can also decrease blood hematocrit and hemoglobin in juvenile rainbow trout. It has been proposed that this CSM-induced anemia results from gossypol binding to iron, rendering it biologically unavailable and leading to hypochromic anemia, and previous research has shown that dietary iron supplementation can alleviate anemia caused by CSM in Nile tilapia [34] and parrot fish, *Oplegnathus fasciatus* [56]. Dietary yeast supplementation restored the blood hematocrit and hemoglobin in the present study. Dietary yeast supplementation effectively restored blood hematocrit and hemoglobin levels in the context of consuming CSM without affecting plasma or liver iron and gossypol levels. The study found no significant differences in hematocrit and hemoglobin between treatments with and without added iron, suggesting that the benefits of yeast are independent of hepatic iron reserves. Additionally, since there were no changes in blood MCH and MCHC across the treatments, the observed declines in hematocrit and hemoglobin levels in the CSM groups may indicate hemodilution, warranting further investigation.

Furthermore, the present findings indicate that dietary yeast supplementation—regardless of whether it was iron-supplemented—did not significantly affect gossypol retention in the fish liver. Previous studies have reported that *S. cerevisiae* has considerable potential to reduce gossypol concentrations in CSM under in vitro fermentation conditions [30]. Accordingly, it can be speculated that yeasts may not effectively neutralize gossypol within the intestinal environment of fish. Variations in the yeast-to-substrate ratio and initial gossypol concentrations may also have influenced these outcomes, warranting further investigation.

The antioxidant system plays a crucial role in fish welfare, immunity, and disease resistance [57, 58]. High levels of CSM in fish diets have been linked to oxidative stress in the liver. For instance, a study on Ussuri catfish, *Pseudobagrus ussuriensis*, found that a diet containing 33%–40% CSM resulted in decreased activities of GPx and TAC, along with an increase in MDA concentration in the fish liver [59]. Similarly, feeding grass carp, *Ctenopharyngodon idella*, a diet containing 49% CSM did not alter antioxidant enzyme levels but did lead to a significant rise in hepatic MDA concentration [60]. In contrast, the current study revealed that dietary CSM did not adversely affect the hepatic antioxidant status of the fish, suggesting a healthy liver. This finding is further supported by the histopathological results (discussed in the following). The discrepancies between studies may be attributed to the lower level of CSM used in our experiment. Notably, both enriched and nonenriched yeast supplementation increased TAC and ascorbate levels in the fish liver, suggesting enhanced reserves of antioxidant molecules. Research has shown that certain components of yeast, such as glucans [61, 62] and nucleotides [63, 64], possess antioxidant properties. Dietary yeast supplementation has also been reported to enhance antioxidant capacity in fish [65, 66]. Therefore, the inclusion of yeast—regardless of enrichment—may have contributed to increased dietary antioxidant compounds, resulting in elevated antioxidant reserves in the fish.

The liver serves as the primary organ for gossypol deposition in fish, and there is a documented relationship between dietary and hepatic gossypol levels and associated histopathological damage. For example, a study on common carp, *C. carpio*, revealed that when dietary free gossypol levels ranged from 306 to 690 mg/kg, hepatic free gossypol levels were measured at 102–198 µg/g, accompanied by moderate to severe fat deposits in the liver. Conversely, when the diet contained only 153 mg/kg of free gossypol, no hepatic gossypol deposition occurred, and only mild fat droplets were observed in hepatocytes [67]. Another investigation on the same species indicated that a diet with 27% CSM caused no lesions in the liver despite a hepatic free gossypol level of 378 µg/g; however, the levels exceeding 45% resulted in a retention of 644 µg/g free gossypol along with hepatocyte shrinkage and hepatic hyperemia [68]. Based on these findings, the free gossypol levels in our study were sufficiently low to prevent significant hepatic deposition, which likely accounted for the absence of notable liver lesions. This also explains the lack of changes in plasma ALT and AST activities, as these enzymes typically increase in the bloodstream when hepatocytes are damaged [69].

In conclusion, the current study demonstrated that incorporating 15% CSM into the diet of rainbow trout (with free gossypol levels ranging from 112 to 131 mg/kg) does not negatively impact fish growth performance, feed efficiency, antioxidant defense, or hepatic histology. However, it does lead to a decrease in blood hematocrit and hemoglobin levels. This reduction may be attributed to hemodilution, as measurements of MCH, MCHC, plasma iron, and both liver iron and gossypol do not indicate hypochromic anemia. Additionally, while yeast supplementation—regardless of iron enrichment—did not enhance fish growth performance, feed efficiency, and hepatic histology in CSM-containing diets, it effectively restored blood hematocrit and hemoglobin levels and increased hepatic antioxidant reserves. Based on these findings, it is advisable to include 15% CSM and 1 × 10^8^ cfu/g of *S. cerevisiae* (PTCC 5052) in the diet of rainbow trout, particularly during their growth from ~31 g to around 125 g. Additionally, further research is encouraged to explore the long-term effects of dietary CSM and other yeast strains, which could enhance our understanding and application of CSM and probiotics in trout nutrition.

## Figures and Tables

**Table 1:** Feed ingredients and dietary composition of diets.

Ingredients^a^	Control	CSM	CSMY	CSMYFE
Wheat flour	234	235	235	235
Soybean meal (defatted)^b^	250	120	120	120
Fishmeal^c^	164	173	173	173
Cottonseed meal^d^	0	150	150	150
Poultry slaughterhouse byproducts^e^	250	250	250	250
Plant oils^f^	83	52	52	52
Mineral premix^g^	10	10	10	10
Vitamin premix^h^	5	5	5	5
Lysine^i^	0.25	0.25	0.25	0.25
Methionine^i^	0.15	0.15	0.15	0.15
Chemical composition^a^				
Moisture	95	98	92	94
Crude protein	414	417	416	414
Crude fat	170	179	175	172
Crude ash	77	70	72	75
Crude fiber	17.5	34.8	33.6	34.2
Phosphorous	0.66	0.72	0.75	0.70
Total gossypol (mg/kg)	nd	1660	1710	1600
Free gossypol (mg/kg)	nd	121	112	130
Iron (mg/kg)	152	153	148	151

^a^Amount as g/kg, otherwise stated.

^b^Crude protein, 42.5%; and crude fat, 0.5%.

^c^Kilka fishmeal; crude protein, 727 g/kg; and crude fat, 96 g/kg.

^d^Crude protein, 323 g/kg; crude fat, 272 g/kg; crude ash, 35 g/kg; crude fiber, 134 g/kg.

^e^Crude protein, 521 g/kg; and crude fat, 229 g/kg.

^f^Mixture of canola and sunflower oils at a ratio of 2:1.

^g^Amineh Gostar Co. (Tehran, Iran). The premix provided the following amounts of vitamin to the diets (per kg): A, 1600 IU; D3, 500 IU; E, 20 mg; K, 24 mg; B3, 12 mg; B5, 40 mg; B2, 10 mg; B6, 5 mg; B1, 4 mg; H, 0.2 mg; B9, 2 mg; B12, 0.01 mg; C, 60 mg; inositol, 50 mg.

^h^Amineh Gostar Co. (Tehran, Iran). The premix provided the following amounts of minerals to the diets (per kg): Se, 0.15 mg; Fe, 2.5 mg; Co, 0.04 mg; Mn, 5 mg; iodate, 0.05 mg; Cu, 0.5 mg; Zn, 6 mg; choline, 150 mg.

^i^CheilJedang Co., Seoul, Korea.

**Table 2:** Amino acid (% of diet) and fatty acid (% of dietary fat) profiles of diets.

Analyzed items	Control	CSM	CSMY	CSMYFE
*Amino acids*				
Arg	2.8	2.9	2.8	2.7
Gly	2.8	2.8	2.9	2.8
Ser	2.1	2	2	2.2
His	1	1	1.1	1.1
Ile	1.6	1.5	1.6	1.4
Leu	2.4	2.2	2.4	2.3
Lys	2.5	2.5	2.6	2.5
Met	1	1	1.1	1
Cis	0.52	0.76	0.62	0.71
Phe	1.7	1.7	1.8	1.7
Tyr	1.1	0.94	0.9	1
Thr	1.5	1.4	1.4	1.5
Trp	0.48	0.46	0.45	0.48
Val	1.9	1.9	2	1.9
*Fatty acids*				
C14:0	0.67	0.86	0.80	0.77
C14:1n5	0.12	0.07	0.05	0.08
C16:0	19.9	21.2	22	21
C16:1n7	2.5	2.4	2.6	2.3
C18:0	6.8	5.9	6	5.7
C18:1n9	34	31	30.3	31.3
C18:1n7	1.5	0	0	0
C18:2n6*cis*	26.7	32	32.3	31.5
C18:3n3	2.4	1.6	1.8	1.7
C20:0	0.48	0.42	0.38	0.41
C20:1n9	0.77	0.56	0.60	0.54
C20:2n6	0.16	0.11	0.13	0.14
C20:4n6	0.49	0.42	0.38	0.45
C20:3n3	0	0.04	0.06	0.03
C20:5n3	0.65	0.63	0.65	0.62
C22:0	0.33	0.24	0.22	0.21
C22:1n9	0.31	0.09	0.10	0.08
C22:6n3	2.4	2.3	2.4	2.5
C24:0	0.50	0.21	0.23	0.20

**Table 3:** Growth performance and survival of rainbow trout in the control, CSM, CSMY, and CSMYFE treatments (mean ± SD; *n* = 4).

Tested parameters	Control	CSM	CSMY	CSMYFE	Sig.
Initial weight (g)	31.6 ± 0.10	31.3 ± 0.60	31.8 ± 0.17	31.6 ± 0.00	0.234
Final weight (g)	121 ± 4.49	126 ± 4.53	125 ± 3.74	123 ± 2.39	0.336
Specific growth rate (%/day)	2.45 ± 0.07	2.53 ± 0.03	2.50 ± 0.05	2.47 ± 0.04	0.123
Weight gain (%)	284 ± 14.8	303 ± 7.04	295 ± 10.6	289 ± 7.55	0.123
Feed conversion ratio	1.00 ± 0.03	0.95 ± 0.02	1.01 ± 0.05	0.98 ± 0.01	0.107
Survival (%)	100	100	96.7 ± 3.85	100	0.073

**Table 4:** Hematological parameters of rainbow trout in the control, CSM, CSMY, and CSMYFE treatments.

Tested parameters	Control	CSM	CSMY	CSMYFE	Sig.
Erythrocyte (10^6^/µL)	1.39 ± 0.16	1.17 ± 0.20	1.55 ± 0.17	1.76 ± 0.38	0.065
Hematocrit (%)	40.5 ± 3.11^b^	33.3 ± 3.59^a^	41.0 ± 2.58^b^	43.8 ± 4.03^b^	0.005
Hemoglobin (g/dL)	7.90 ± 0.71^b^	6.45 ± 0.70^a^	8.58 ± 0.56^b^	8.88 ± 0.79^b^	0.002
Mean corpuscular volume (fL)	294 ± 16.9	289 ± 18.1	266 ± 13.2	254 ± 36.3	0.150
Mean corpuscular hemoglobin (pg)	57.2 ± 2.69	55.8 ± 4.44	55.7 ± 2.95	54.4 ± 6.86	0.575
Mean corpuscular hemoglobin concentration (g/dL)	19.5 ± 1.14	19.4 ± 0.88	20.9 ± 0.48	20.3 ± 0.80	0.090
Leukocyte (10^3^/µL)	12.7 ± 1.07	9.25 ± 4.67	8.17 ± 1.17	8.93 ± 2.69	0.158
Lymphocyte (%)	93.8 ± 0.50	92.5 ± 1.00	91.8 ± 1.50	92.3 ± 1.50	0.159
Neutrophil (%)	4.00 ± 0.82	5.00 ± 0.82	5.50 ± 1.29	5.25 ± 0.96	0.207
Monocyte (%)	2.25 ± 0.50	2.50 ± 0.58	2.75 ± 0.50	2.50 ± 0.58	0.644

*Note:* Different superscript letters within a row show significant differences among the treatments (mean ± SD; *n* = 4).

**Table 5:** Plasma/hepatic iron and gossypol levels of rainbow trout in the control, CSM, CSMY, and CSMYFE treatments.

Tested parameters	Control	CSM	CSMY	CSMYFE	Sig.
Plasma iron (µg/dL)	510 ± 67.3	610 ± 269	690 ± 159	458 ± 256	0.418
Hepatic iron (mM/g ww)	0.54 ± 0.05^a^	0.53 ± 0.07^a^	0.56 ± 0.05^a^	0.74 ± 0.07^b^	0.001
Hepatic gossypol (µg/g ww)	0.00^a^	39.0 ± 7.07^b^	41.0 ± 8.29^b^	38.3 ± 8.88^b^	<0.001

*Note:* Different superscript letters within a row show significant differences among the treatments (mean ± SD; *n* = 4).

**Table 6:** Hepatic antioxidant-related parameters of rainbow trout in the control, CSM, CSMY, and CSMYFE treatments (mean ± SD; *n* = 4).

Tested parameters	Control	CSM	CSMY	CSMYFE	Sig.
Superoxide dismutase (U/mg pr)	33.0 ± 5.72	37.5 ± 6.61	38.8 ± 4.50	36.8 ± 4.42	0.496
Catalase (U/mg pr)	56.8 ± 5.38	57.8 ± 4.79	55.5 ± 4.80	56.5 ± 4.93	0.935
Reduced glutathione (µM/g ww)	2.48 ± 0.53	3.40 ± 0.43	2.95 ± 0.62	3.28 ± 0.51	0.111
Malondialdehyde (nM/g ww)	51.8 ± 3.59	50.5 ± 6.19	51.8 ± 5.91	50.0 ± 4.16	0.945

**Table 7:** Plasma antioxidant parameters and aminotransferase activities of rainbow trout in the control, CSM, CSMY, and CSMYFE treatments.

Tested parameters	Control	CSM	CSMY	CSMYFE	Sig.
Total antioxidant capacity (nM/L)	0.17 ± 0.04^a^	0.18 ± 0.05^a^	0.25 ± 0.04^b^	0.30 ± 0.03^b^	0.002
Ascorbate (mg/dL)	2.45 ± 0.31^a^	2.50 ± 0.41^a^	3.28 ± 0.49^b^	3.30 ± 0.54^b^	0.025
Alanine aminotransferase (U/L)	16.3 ± 3.86	17.5 ± 3.87	15.8 ± 3.30	15.5 ± 4.20	0.883
Aspartate aminotransferase (U/L)	138 ± 19.7	135 ± 17.2	137 ± 24.3	139 ± 18.6	0.993

*Note:* Different superscript letters within a row show significant differences among the treatments (mean ± SD; *n* = 4).

## Data Availability

Data are available upon reasonable request from the corresponding author.
